# Variant of the Anconeus Epitrochlearis Muscle: A Case Report

**DOI:** 10.7759/cureus.3201

**Published:** 2018-08-24

**Authors:** Chrissie Massrey, Joe Iwanaga, Basem Ishak, Rod J Oskouian, Marios Loukas, R. Shane Tubbs

**Affiliations:** 1 Seattle Science Foundation, Seattle, USA; 2 Medical Education and Simulation, Seattle Science Foundation, Seattle, USA; 3 Neurosurgery, Seattle Science Foundation, Seattle, USA; 4 Neurosurgery, Swedish Neuroscience Institute, Seattle, USA; 5 Anatomical Sciences, St. George's University, St. George's, GRD

**Keywords:** anconeus epitrochlearis, variant, cubital tunnel syndrome

## Abstract

The anconeus epitrochlearis is a muscle variant sometimes present at the elbow. It is present in up to 34% of individuals and has been implicated in some cases of cubital tunnel syndrome. We report an unusual variant of this muscle with additional proximal attachments in the arm. We will review and discuss the background and the clinical relevance of such a muscle.

## Introduction

The anconeus epitrochlearis muscle is seen in many animal species including: reptiles, amphibians, and mammals [1]. In humans, the muscle is a variant. The anconeus epitrochlearis is also termed the anconeus internus, anconeus parvus, epitrochleo-olecranonis, and epitrochleocubital muscle [2,3]. Its origin is from the medial epicondyle of the humerus and its insertion is onto the olecranon of the ulna [1]. The anconeus epitrochlearis is often thought of as an extension of the triceps brachii, but some have considered it a variant of the flexor carpi ulnaris because it courses over the groove for the ulnar nerve [4] and is innervated by the ulnar nerve [5]. When present, the anconeus epitrochlearis forms the roof of the cubital tunnel [6]. The muscle tends to course obliquely and backward [6], tightens when the elbow joint is flexed, and becomes relaxed when the elbow is extended [7]. Evolutionarily, it was thought to have been a weak extensor of the elbow, but over time has evolved to be Osborne’s ligament of the elbow [5].

There has been little documentation on variations in the anatomy of the anconeus epitrochlearis [5]; however, Macalister and Gruber were able to observe slight differences in the muscle. They found the muscle could be narrow or wide, one head or two heads, tendinous or fleshy, and sometimes shaped like a triangle, but more often flat and shaped like a rectangle [8,9]. In the case presented here, the muscle had proximal extensions into the triceps brachii and medial intermuscular septum.

## Case presentation

**Figure 1:**
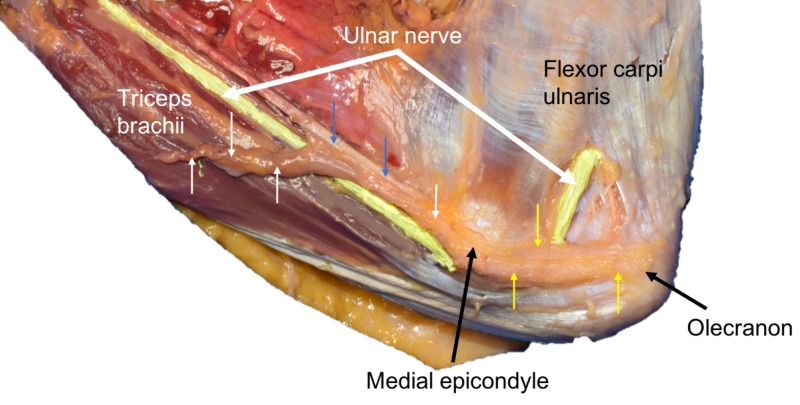
Left elbow of the case presented herein. Note the ulnar nerve (colored yellow) coursing from the medial arm into the cubital tunnel and deep to the anconeus epitrochlearis where it attaches (yellow arrows) the medial epicondyle of the humerus to the olecranon. Particular to this case is the proximal extension of this variant muscle onto the medial intermuscular septum (blue arrows) and superficial to the triceps brachii muscle (short white arrows to far left).

During the routine dissection of the left medial elbow of an adult female fresh frozen cadaver aged 89 years at death, an anconeus epitrochlearis muscle was identified. The innervation of the muscle was via the ulnar nerve. The muscle had its typical course between the olecranon and medial epicondyle. However, proximal to the medial epicondyle, the muscle had muscular extensions that doubled the overall length of the muscle. Proximally, the muscle had a direct connection to the medial intermuscular septum anteriorly and posteriorly, it attached into the triceps brachii muscle (Figure 1). No other musculoskeletal or neurovascular anatomical variants were noted on the ipsilateral or contralateral sides.

## Discussion

We identified a variant of the anconeus epitrochlearis muscle, that to our knowledge has not been previously reported. The anconeus epitrochlearis, which is found in 1–34% of individuals [8-13], was first described by Gruber in 1866 [9]. Gruber investigated the presence of the anconeus epitrochlearis muscle using 100 cadavers. He found the muscle in 26 males and eight females and that it was bilateral in 15 males and four females [6]. Furthermore, a study done by Nascimento and Ruiz [1] found 29% of individuals have this muscle variation, and it was not associated with age or sex.

The presence of this muscle has clinical relevance in relation to the cubital tunnel syndrome. Some speculate it may be a cause of cubital tunnel syndrome, and others have suggested it is actually protective for the ulnar nerve [1]. Cubital tunnel syndrome is a common cause of ulnar nerve entrapment in the upper extremity. The clinical signs of this syndrome are sensory loss or hypoesthesia in the fourth and fifth digits and loss of strength in finger abduction [14]. Wilson et al. performed a retrospective cohort study to understand the relationship between the anconeus epitrochlearis and cubital tunnel syndrome. The authors hypothesized that the risk of developing cubital tunnel syndrome may be reduced if Osborne’s ligament is replaced with the anconeus epitrochlearis, which is more flexible [6]. These researchers also hypothesized that if an individual did have both cubital tunnel syndrome and an anconeus epitrochlearis muscle present, the pathology would be secondary to a hypertrophied muscle [6]. Wilson et al. found that the muscle appeared more frequently in individuals who were asymptomatic (15.5%) than those who had undergone surgical intervention to correct symptomatic cubital tunnel syndrome (5.4%) [6].

Fernandez et al. [14] reported six cases with ulnar nerve entrapment at the elbow with presence of the anconeus epitrochlearis. All six patients required surgery to relieve their symptoms.

## Conclusions

As the anconeus epitrochlearis can be involved in patients with cubital tunnel syndrome, its anatomical variations, such as the one presented herein, should be considered by the clinician treating such patients.
